# Wilson’s Disease Presenting as Resistant Rickets

**DOI:** 10.4021/gr262w

**Published:** 2011-01-20

**Authors:** Jagdish P Goyal, Nagendra Kumar, Sanjeev S Rao, Vijay B Shah

**Affiliations:** a Department of Pediatrics, Govt Medical College, Surat, India

**Keywords:** Wilson’s disease, Rickets

## Abstract

Wilson’s disease is most common disorder of cooper metabolism. It has varied clinical presentations. We report a 12 years old female child presenting with genu valgum progressed over 6 months. Careful examination, high index of suspicion and investigations confirmed Wilson’s disease.

## Introduction

The first reported case of Wilson’s disease with rachitic presentation was in 1968 by Cavallino [1]. Since then only few pediatric cases of Wilson’s disease with this rare presentation have been reported.

## Case Report

A 12 years old female child was admitted with mild pain and deformity at both knee joints which was progressive over 6 months. There was no history of swelling or morning stiffness of joints, no history of repeated fractures or similar illness in family. She was admitted for jaundice 3 years back. She was given mega doses of vitamin D before she referred to our center.

**Figure 1:**
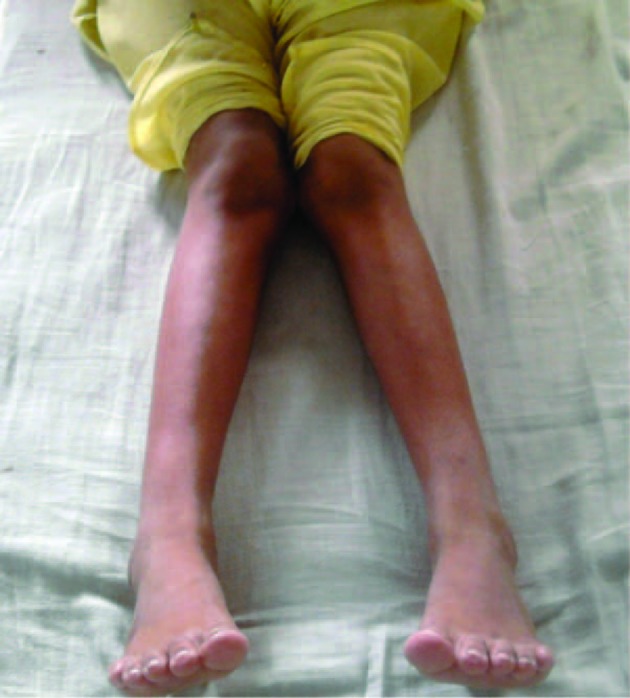
Genu valgam with increased bimalleolar distance.

On examination she was normally built and nourished with height 138 cm and weight 27 kg, and vital signs and parameters were normal. On Local examination, knee joint was normal and there was genu valgum with bimalleolar distance of 13 cm; angulations were more in right than left (Fig. 1). Rest of systemic examination was found to be normal.

Investigations revealed Hb10.4 gm/dl, TLC 4.8 x 10^3^/µL, PC 208 x 10^3^/µL, ESR 25 mm/ h, ALT 30 U/L, AST 22 U/L, bilirubin 0.8 mg/dl, Urea 28 mg/dL, Cratinine 1.1 mg/dL, Na 149 mEq/L, K 3.1 mEq/L, Ca 7.5 mg/dL, Phosphate 4.8 mg/dL (2.9 - 5.4), AlkPO_4_ 203 units/L (105 - 420). X-ray local part (knee) was suggestive of mild osteoporotic changes.

Urine examination showed urine pH 7.5, urine anion gap +26, urinary calcium 700mg (100 - 250) and urinary phosphate 300mg (900 - 1300). ABG revealed pH 7.29, PO_2_ 108 mmHg, PCO_2_ 36 mmHg, and HCO_3_ 17 mEq/L with anion gap of 14. Parathormone level was 25.5 pg/ml (12 - 95) and Vitamin D_3_ level was 27 ng/ml (5.9 - 59).

We suspected Wilson’s disease in this unexplained bone disease. Slit lamp examination of her eyes revealed Keyser-Fleisher ring. Ceruloplasmin level was 2.81 mg/dL (25 - 60). Urinary copper excretion was 1140 µg/day. USG abdomen revealed diffuse fatty infiltration of liver. MRI brain showed copper deposits in caudate nucleus and putamina. So diagnosis of Wilson’s disease with rickets due to renal tubular acidosis was made. She was given zinc acetate, D-penicillamine, sohl solution and oral calcium; and asked to follow up in OPD to observe improvement.

## Discussion

Wilson's disease (WD; Hepatolenticular degeneration) is a rare inherited autosomal recessive inborn error of copper metabolism characterized by toxic accumulation of copper in liver, brain, cornea and other tissues [2]. The genetic basis for WD is linked to one of the transport proteins. It was linked to the long arm of chromosome 13 by identifying the association of WD with esterase D deficiency in an inbred Arab family [3]. Subsequent analysis revealed a defect in a P-type adenosine triphosphatase (ATPase) involved in the transport of copper across the trans-Golgi and into transport vesicles [4]. The interesting feature of WD is its wide variety of phenotypic presentations which is also influenced by race and region. This may due to genetic heterogeneity and different types of mutations involved.

Clinical presentation of WD in India does not always follow the pattern as is usually seen in the Western world [5, 6]. Wadia et al [7] first drew attention in this regard and observed that generalized osteoporosis, rickets, or even renal rickets could be the striking manifestations, at least in 8 out of 23 patients. Rickets and osteoporosis as presenting feature is also reported by few other Indians authors [8, 9].

In our case we also found rickets as only presentation. The child had no symptoms and signs related to liver and CNS involvement although involvement was detected through MRI brain and USG abdomen. There are many mechanisms implicated for rickets in WD. It may be due to hypoparathyroidism, and renal rickets may be due to fanconi disease. In our case, we ruled out hypoparathyroidism, and rickets was probably due to renal tubular acidosis.
